# Long-Term Functional and Cytoarchitectonic Effects of the Systemic Administration of the Histamine H_1_ Receptor Antagonist/Inverse Agonist Chlorpheniramine During Gestation in the Rat Offspring Primary Motor Cortex

**DOI:** 10.3389/fnins.2021.740282

**Published:** 2022-01-24

**Authors:** Rocío Valle-Bautista, Berenice Márquez-Valadez, Gabriel Herrera-López, Ernesto Griego, Emilio J. Galván, Néstor-Fabián Díaz, José-Antonio Arias-Montaño, Anayansi Molina-Hernández

**Affiliations:** ^1^Departamento de Fisiología, Biofísica y Neurociencias, Centro de Investigación y de Estudios Avanzados del Instituto Politécnico Nacional, Ciudad de México, Mexico; ^2^Laboratorio de Investigación en Células Troncales y Biología del Desarrollo, Departamento de Fisiología y Desarrollo Celular, Subdirección de Investigación Biomédica, Instituto Nacional de Perinatología Isidro Espinosa de los Reyes, Ciudad de México, Mexico; ^3^Departamento de Farmacobiología, Centro de Investigación y de Estudios Avanzados del Instituto Politécnico Nacional, Ciudad de México, Mexico

**Keywords:** chlorpheniramine, cerebral cortex development, cytoarchitecture, hitamine H_1_ receptor, pregnancy, cortical function

## Abstract

The transient histaminergic system is among the first neurotransmitter systems to appear during brain development in the rat mesencephalon/rhombencephalon. Histamine increases FOXP2-positive deep-layer neuron differentiation of cortical neural stem cells through H_1_ receptor activation *in vitro*. The *in utero* or systemic administration of chlorpheniramine (H_1_ receptor antagonist/inverse agonist) during deep-layer cortical neurogenesis decreases FOXP2 neurons in the developing cortex, and H_1_R- or histidine decarboxylase-knockout mice show impairment in learning and memory, wakefulness and nociception, functions modulated by the cerebral cortex. Due to the role of H_1_R in cortical neural stem cell neurogenesis, the purpose of this study was to evaluate the postnatal impact of the systemic administration of chlorpheniramine during deep-layer cortical neuron differentiation (E12–14) in the primary motor cortex (M1) of neonates (P0) and 21-day-old pups (P21). Chlorpheniramine or vehicle were systemically administered (5 mg/kg, i.p.) to pregnant Wistar rats at gestational days 12–14, and the expression and distribution of deep- (FOXP2 and TBR1) and superficial-layer (SATB2) neuronal cortical markers were analyzed in neonates from both groups. The qRT-PCR analysis revealed a reduction in the expression of Satb2 and FoxP2. However, Western blot and immunofluorescence showed increased protein levels in the chlorpheniramine-treated group. In P21 pups, the three markers showed impaired distribution and increased immunofluorescence in the experimental group. The Sholl analysis evidenced altered dendritic arborization of deep-layer neurons, with lower excitability in response to histamine, as evaluated by whole-cell patch-clamp recording, as well as diminished depolarization-evoked [^3^H]-glutamate release from striatal slices. Overall, these results suggest long-lasting effects of blocking H_1_Rs during early neurogenesis that may impact the pathways involved in voluntary motor activity and cognition.

## Introduction

Histamine (HA) participates in rat central nervous system (CNS) development. A transitory fetal histaminergic system co-localizes with serotonin neurons in the mesencephalon/rhombencephalon at embryo day 12 (E12; Auvinen and Panula, 1988). Also, the highest concentration of HA coincides with higher neurogenesis at E14 and E16 (Vanhala et al., 1994). Four G protein-coupled receptors (H_1_R–H_4_R) mediate HA actions (Panula et al., 2015), and H_1_R and H_2_R are widely distributed in the rat CNS at E14 and E15, respectively (Kinnunen et al., 1998; Heron et al., 2001; Karlstedt et al., 2001).

Through H_1_R activation, HA promotes neuron differentiation of cortical neural stem cells (NSCs) obtained from E14 embryo and adult tissue (Molina-Hernández and Velasco, 2008; Bernardino et al., 2012). In cortical NSCs, HA promotes FOXP2-positive neuron differentiation (Molina-Hernández et al., 2013). FOXP2 (marker of deep-layer neurons) is involved in learning motor skills, and specific mutations are related to verbal dyspraxia in humans and impaired ultrasonic vocalization and singing performance in rodents and birds, respectively. These functional processes are regulated by the cortex, basal ganglia, thalamus, and cerebellum (Burkett et al., 2018; Co et al., 2020; den Hoed et al., 2021). During corticogenesis, FOXP2 participates in the transition from NSCs to intermediate precursors (IP) required for neuron differentiation (Tsui et al., 2013).

The intrauterine administration of the H_1_R antagonist/inverse agonist chlorpheniramine into the ventricular lumen of E12, when the H_1_R is highly expressed in rats, results in decreased immunoreactivity to βIII-tubulin (a marker for immature neurons) and FOXP2 in the cortical neuroepithelium at E14 (Molina-Hernández et al., 2013).

Embryos from diabetic rats and mice show increased neurogenesis (Fu et al., 2006; Solís et al., 2017), which in the rat is prevented by the systemic administration at E12 of chlorpheniramine, highlighting the relevance of the H_1_R on neurogenesis.

Moreover, H_1_R or histidine decarboxylase (the limiting histamine-producing enzyme) knockout mice show impairment in brain functions such as learning and memory, wakefulness, and nociception (Mobarakeh et al., 2005; Chepkova et al., 2012; Ambree et al., 2014; Parmentier et al., 2016), functions modulated by the motor cerebral cortex (Richardson et al., 2006; Hirano et al., 2018; Poulet and Crochet, 2019; Gamal-Eltrabily et al., 2021).

The motor area of the cerebral cortex is located in the frontal lobe, where the transcription factors serving as layers II/III and V markers are Satb2 and Cux1, whereas Tbr1, Ctip2, and FoxP2 are markers of layers V/VI (Greig et al., 2013; Jabaudon, 2017; Kast et al., 2019). The main function of this cortical region is to modulate movement execution by processing the information received from the ventrolateral thalamic nucleus and sending afferents to the cerebellum, the basal ganglia, and the internal segment of the globus pallidus. Projections from the primary motor cortex (M1) terminate almost entirely in the dorsolateral and central region of the putamen, a nucleus of the basal ganglia. The frontal cortex and the basal ganglia operate together to execute movements and modulate the behaviors that lead to this execution, including emotions and motivation, cognition, and planning (Haber, 2016). At the cytoarchitectonic level, V/VI layers represent more than 50% of the M1 area (Skoglund et al., 1997).

Based on H_1_R effect on deep-layer neurogenesis and the participation of FOXP2 in cortical development and movement, here we studied the long-term effect of chlorpheniramine administration to pregnant rats (5 mg/kg ip; Naranjo and de Naranjo, 1968) during cortical neurogenesis (E12–E14) on the M1 of neonatal (P0) and infant (21-day-old; P21) offspring.

Through molecular, biochemical, morphological, and electrophysiological analyses, this study revealed long-lasting chlorpheniramine effects on FOXP2, SATB2, and TBR1 expression and distribution; reduced dendritic arborizations and neuronal excitability of M1 deep-layer neurons and decreased depolarization-evoked [^3^H]-glutamate release from striatal slices.

## Materials and Methods

All experiments were performed according to the “Guide for the care and use of laboratory animals” (NIH No. 80-23, revised in 1978) and *Norma Oficial Mexicana para la Producción, Cuidado y Uso de Animales de Laboratorio* (NOM-062-ZOO-1999). The experimental protocol was approved by the Institutional Committee for the Care and Use of Laboratory Animals (CICUAL) and the Research, Ethics and Biosafety Committees of the National Institute of Perinatology (INPer; number 3230-21202-01- 2015).

Wistar rats were maintained at constant temperature (22 ± 2°C), light:dark cycle 12:12 h, relative humidity 40%, and water and food *ad libitum*. Females (230–300 g) were placed with a sexually mature male during the dark phase; the following morning, the presence of sperm was determined in a vaginal smear, and this time was considered as E0.5. Females were separated from males and housed individually throughout the gestation.

At gestation day E12, pregnant rats were randomly assigned to each experimental group (control or chlorpheniramine). From E12 to E14, the chlorpheniramine group received a daily intraperitoneal injection of the antihistamine (5 mg/kg; Sigma-Aldrich, St Louis, MO, United States), while the control group received vehicle (injectable water; Laboratorios PiSA, Guadalajara, Jal., Mexico) (Naranjo and de Naranjo, 1968; Márquez-Valadez et al., 2019).

We recently reported that only male pups from chlorpheniramine-treated rats during pregnancy (same dose and time of administration as in this study) showed a decrease in movement speed, in this work, we therefore use only male offspring (Márquez-Valadez et al., 2019).

Animals of both groups were euthanized by decapitation at P0 (within the first 2 h after birth) and P21. The brain was dissected, and the frontal cerebral cortex was isolated. Fresh tissue was used for electrophysiology, [^3^H]-glutamate release, mRNA expression, Golgi-Cox stain, and radioligand binding assays, while for immunofluorescence, the tissue was fixed by transcardiac perfusion with Boüin’s solution.

### RNA Extraction and qRT-PCR

**FIGURE 1:**
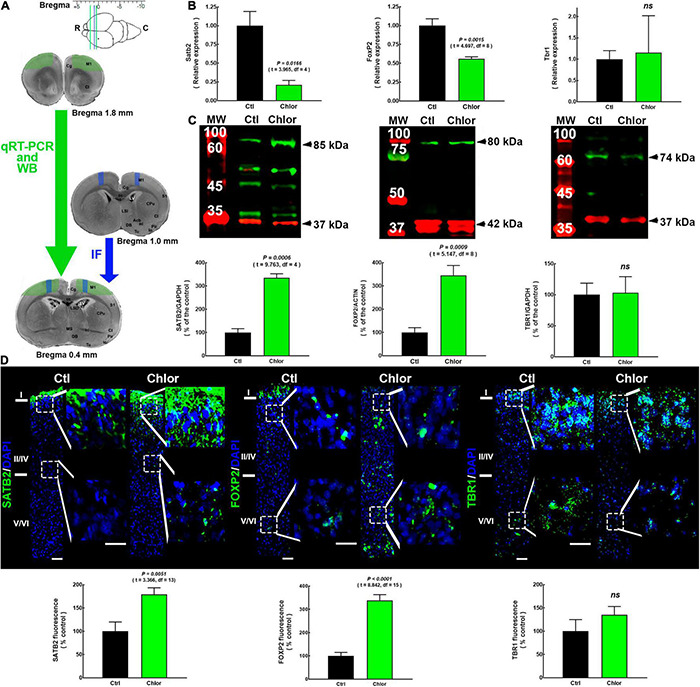
SATB2, FOXP2, and TBR1 expression and distribution in the primary motor cortex of neonates from control and chlorpheniramine-treated dams. **(A)** Upper, image indicating the bregma levels used for qRT-PCR and Western blot (green lines), and immunofluorescence analysis (space between the blue and the caudal green lines). Lower, representative images of coronal brain slices indicate the zones dissected for qRT-PCR by (green area; 1.8–0.4 mm from bregma, as indicated by the green arrow). The blue area indicates the zone where micrographs were taken of slices (1.0–0.4 mm from bregma, blue arrow). R, rostral; C, caudal; IF Immunofluorescence. Modified from Khazipov et al. (2015). **(B)** qRT-PCR analysis of Satb2, FoxP2, and Tbr1. Values were normalized to the relative expression of control animals using the 2^–ΔΔ*CT*^ method and are means ± SEM (n = 3-5; P0). **(C)** Upper, representative Western blots for SATB2 (green, 85 kDa), FOXP2 (green, 80 kDa), TBR1 (green, 74 kDa), and the internal controls GAPDH (red, 37 kDa) or α-actin (red, 42 kDa). Bands on the left lanes are molecular weight ladders (MW). Lower, quantitative fluorometric analysis for SATB2, FOXP2, and TBR1. Values are expressed as percentage of the fluorescence ratio of control values and are means ± SEM (*n* = 3–5; P0). **(D)** Upper, representative M1 reconstructions from five micrographs (10×) of SATB2, FOXP2 and TBR1 (green) immunodetection and DAPI-stained nuclei (blue), at postnatal day zero (P0) from Control (Ctl) and Chlorpheniramine (Chlor) groups (*n* = 3–6; P0). Zoom images (400%) of the dotted white squares of the superficial and deep neocortex are shown on the right of each representative image. Scale bars correspond to 50 and 25 μm for 10× and zoom, respectively. Lower, quantitative analysis of the immunofluorescence of SATB2, FOXP2, and TBR1. Values are expressed as percentage of the fluorescence of controls and are means ± SEM (*n* = 3–6; P0). The statistical analyses in **(A–C)** were performed with unpaired Student’s *t*-test. *ns, non-significant*.

At P0, the frontal cerebral cortices of both hemispheres from one pup per litter were used for expression analysis (n = 3–5 different litters). The dorsal cortex was dissected according to Khazipov et al. (2015), ∼1.8 to 0.4 mm from bregma (Figure 1A).

The frontal cortex was placed in 1 ml of TRIZOL^®^ reagent (Life Technologies, Bartlesville, OK, United States) and stored at −70°C until required. To obtain total RNA, the tissue was homogenized by pipetting, incubated for 15 min at 30°C, chloroform was then added, and samples were vigorously shaken for 15 s and then incubated for 3 min at room temperature. After centrifugation (1,700 × *g*) for 15 min at 4°C, the chloroform phase was transferred to a new tube, isopropanol was added, the tubes were vigorously shaken, and samples were incubated at 30°C for 10 min before centrifugation (11,000 × *g*) at 4°C. The supernatant was then removed, and the pellet was washed twice for 5 min with 1 ml of ethanol.

Total RNA was resuspended in 20 μl of RNAse-free water and incubated for 10 min at 60°C; RNA integrity was determined by the presence of 18S, 28S, and 5S ribosomal RNAs, after the electrophoresis of 1 μl of total RNA in 2% agarose gels.

For reverse transcription (RT) reactions, 1 μg of total RNA was mixed with 0.5 μg of random hexamers, 0.5 μl of a mixture of dinucleotide triphosphates (dNTPs, 10 mM), 2 μl of reverse transcription buffer (10×), 0.5 μl of ribonuclease inhibitor (40 U/μl), 4 μl of MgCl_2_ (25 mM) and 5 U of AMV Retrotranscriptase (Promega, Madison, WI, United States) in a total volume of 20 μl with RNAase-free water. The mixture was incubated for 60 min at 42°C, the reaction was stopped for 5 min at 95°C, and the cDNA was kept at −20°C until required.

End-point PCR was performed to verify the size of the products. Dynamic ranges were performed for each transcript using the KAPA™ master mix Syber Fast qPCR^®^ (KAPA, Bartlesville, OK, United States), 1 μl of pre-amplified cDNA from 1, 10, 100, and 1,000 ng of cDNA to determine detection threshold, efficacy, slope, and to ensure a single fragment of amplification melting curves were obtained to observe a single peak (Kubista et al., 2006).

For qPCR, 1 μl of 500 ng of pre-amplified cDNA was assayed to obtain the relative expression of Satb2, Tbr1, and FoxP2, with GAPDH (glyceraldehyde 3-phosphate dehydrogenase) as an endogenous control. The sequences of the primers employed were those previously reported by Valle-Bautista et al. (2020):

Satb2 Forward (F): 5′-CCGCACACAGGGATTATTGTC-3′; reverse (R): 5′-TCCACTTCAGGCAGGTTGAG-3′, Tbr1 F: 5′-GGAAGTGAATGAGGACGGCA-3′; R: 5′-TGGCGTAGTT GCTCACGAAT-3′, FoxP2 F: 5′-GAAAGCGCGAGACACATCG GAPDH-3′; R: 5′-GAAGCCCCCGAACAACACA-3′, and GAPD H F: 5′-GGACCTCATGGCCTACATGG-3′; R: 5′-CCCCTCCT GTTGTTATGGGG-3′.

Reactions were performed with an initial denaturation period of 2 min at 95°C, followed by 40 denaturation cycles of 30 s at 95°C, alignment for 15 s at 63°C (FoxP2), 60°C (Tbr1), 57°C (Satb2), or 58°C (GAPDH). At the end of the incubation, melting curves were obtained. Results were analyzed using the 2^–ΔΔ*CT*^ relative expression method (Livak and Schmittgen, 2001).

### Immunofluorescence

One or two animals (P0 and P21) per litter were used (*n* = 3–6 different litters). P0 and P21 rats received 50 mg/kg of pentobarbital intraperitoneally (Laboratorios PiSA) and were transcardiacally perfused with phosphate-buffered saline (PBS), pH 7.4, followed by Boüin’s solution (15: 15: 1; aqueous solution saturated with picric acid: 40% formalin: glacial acetic acid) at room temperature and flow rate 0.5 ml/min for P0 and 00.7 ml/min for P21 using an infusion pump (Model 07525-40, Masterflex, Gelsenkirchen, Germany). The brain was removed and immersed for 3 days in Boüin’s solution and 30% sucrose solution for three additional days. Once fixed and cryoprotected, the brain was frozen, and coronal slices (10 μm thick) were obtained with a cryostat (Leica CM1850 UV, Wetzlar, Germany) and recovered on slides treated with poly-L-lysine (Sigma-Aldrich).

**FIGURE 2:**
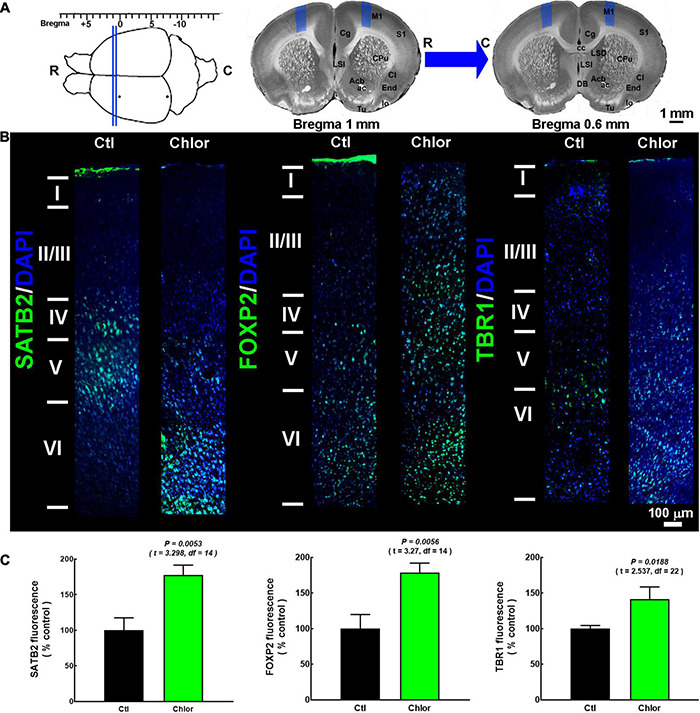
SATB2, FOXP2, and TBR1 distribution in the primary motor cortex of 21-day-old offspring from control and chlorpheniramine-treated dams. (**A**, Upper left) The image shows the coordinates (from bregma) used for immunofluorescence analysis (between the blue lines). (Upper right) The blue areas represent the zone where micrographs were taken of slices (1.0–0.6 mm from bregma, as indicated by the blue arrow). R, rostral; C, caudal. Modified from Khazipov et al. (2015). **(B)** Representative M1 reconstruction from seven micrographs (10×) of SATB2, FOXP2, and TBR1 (green) immunodetection and DAPI-stained nuclei (blue), at postnatal day 21 in the primary motor cortex from Control (Ctl) and chlorpheniramine-treated (Chlor) groups. Images are representative from 3–6 P21 animals. **(C)** Quantitative fluorometric analysis. Values are expressed as a percentage of the fluorescence obtained in the controls and are means ± SEM from 3–6 P21 animals. The statistical analysis was performed with unpaired Student’s *t*-test.

Four coronal slices (10 μm thick) containing the M1 were recovered every 30 μm containing slices at ∼1–0.4 mm from Bregma at P0 (Figure 1A), and ∼1.0–0.6 mm for P21, according to Khazipov et al. (2015) (Figure 2A).

The sections were rinsed with PBS and incubated for 1 h with the blocking and permeabilization solution (PBS with 10% normal goat serum and 0.3% Triton X-100). The autofluorescence was blocked with TrueBlack (Biotium, Hayward, CA, United States), and the tissue was incubated overnight at 4°C with primary antibodies (Table 1) diluted in PBS containing 10% normal goat serum. The following day the tissue was washed three times (10 min each) with PBS at room temperature and then incubated for 1 h at room temperature with the corresponding secondary antibodies (Table 1). Nuclei were stained with DAPI (4′, 6-diamidino-2-phenylindole; 1 μg/ml) for 10 min. After washing three times for 10 min with PBS, the tissue was mounted with Aqua Poly/mount (Polyscience, Warrington, PA, United States).

**TABLE 1:** Primary and secondary antibodies.

Antibody	Description	Dilution	Specie	Supplier and catalog
Reelin	Glycoprotein secreted by Cajal-Retsius neurons. Stop signal for neuronal migration, localized to the marginal zone or layer I during embryo and early postnatal development, respectively	1:250 (IF)	Mouse	Millipore MAB5366
SATB2	Transcription factor, marker of cortical layers II, III, and IV	1:250 (IF) 1:1500 (WB)	Rabbit	Abcam Ab34735
TBR1	Transcription factor, marker of cortical layers V and VI	1:100 (IF) 1:500 (WB)	Rabbit	Abcam Ab31940
FOXP2	Transcription factor marker of cortical layers V y VI	1:250 (IF) 1:100 (WB)	Rabbit	Abcam Ab16046
GAPDH	Internal control (WB)	1:1,500 (WB)	Mouse	GeneTex GTX41027
β-actin	Internal control (WB)	1:1,500 (WB)	Mouse	GeneTex GTX82559
Alexa Fluor 488	Secondary antibody	1:1,000 (WB)	Mouse Rabbit	Life Technologies A-11034 A-11029
Alexa Fluor 568	Secondary antibody	1:1,000 (IF)	Mouse Rabbit	Life Technologies A-11011 A-11004
IRDye 680RD	Secondary antibody	1:10,000 (WB)	Mouse Rabbit	LI-COR 926-68072 926-68073
IRDye 680RD	Secondary antibody	1:10,000 (WB)	Mouse Rabbit	LI-COR 926-32212 926-32213

Micrographs of M1 were obtained with a charge-coupled device camera (ORCA-Flash 2.8 CCD model C11440; Hamamatsu, Honshu, Japan) coupled to the epifluorescence microscope (Olympus IX-81, Shinjuku, Tokyo, Japan). Five (P0) or seven (P21) focal planes from the pial surface to the lateral ventricle were captured per slice (10×) to reconstruct the entire M1 area using Adobe Photoshop software (C6S; San Jose, CA, United States). The exposition and gain values used for the first micrograph from a control sample for each marker were maintained for all subsequent acquisitions. Fluorescence quantification was performed with Fiji software (Schindelin et al., 2012), and data were expressed as percentage of the fluorescence obtained in control samples.

### Western Blot

The frontal cortices of both hemispheres were dissected from one P0 pup from different litters to obtain total protein extracts (Figure 1A). Three to five different animals from different litters per group (n = 3–5 different litters) were used.

The tissue was homogenized in lysis solution (25 mM Tris-HCl, pH 7.4, 1% IGEPAL, 100 mM NaCl) containing protease inhibitors (Amresco, Solon, OH, United States), the lysate was centrifuged at 13,000 × *g* for 10 min at 4°C, the supernatant was recovered, and the protein content was quantified by the Bradford method (Sigma-Aldrich; Bradford, 1976).

For electrophoresis, 20–80 μg of protein per sample and 2.5 μl of the molecular weight marker (Precision Plus Protein™ WesternC™ Blotting Standards, Bio-Rad or Trident Prestained Protein Ladder, GeneTex) were loaded on 10% polyacrylamide (SDS-PAGE) gels and proteins separated for 30 min at 70 V (4% separator gel) and 2 h at 100 V (10% concentrator gel), using the MiniProtean II system (Bio-Rad, Hercules, CA, United States). Proteins were then transferred to nitrocellulose membranes (Amersham™ Hybond™-ELC, Buckinghamshire, United Kingdom), using the Trans-Blot^®^ semi-wet transfer system (Bio-Rad; Villanueva, 2008) at 1.3 A/25 V for 12 min.

Non-specific binding of antibodies was blocked by incubating (1 h) with 7% fatty acid-free milk (SVELTY^®^ Milk, Nestlé, Mexico City, Mexico) in TBS buffer (20 mM Tris-base, 150 mM NaCl, pH 7.4). Membranes were incubated overnight at 4°C with the primary antibodies (Table 1) diluted in TBS, washed with TBS-T solution (TBS containing 0.1% Tween-20), and then incubated with secondary antibodies (Table 1) for 1 h at room temperature. Bands corresponding to the proteins of interest were detected by scanning the membranes on the Odyssey Clx system and analyzed with the Image Studio software version 4.0 (Li-COR Bioscience, Lincoln, NE, United States). The fluorescence signals obtained for GAPDH or α-actin were used as internal controls.

### Dendritic Complexity and Sholl Analyses

Golgi-Cox staining was performed to evaluate the structure and orientation of pyramidal neurons in the M1 from P21 animals (FD Rapid GolgiStain Kit, FD Neurotechnologies, Columbia, MD, United States). Brains from 3 or 4 animals from different litters (*n* = 3–4) were obtained after decapitation, washed with PBS at 4°C, and after removal of blood vessels and meninges cut into two coronal segments placed in the impregnation solution for 12 h, and stored protected from the light in a new impregnation solution for two weeks at room temperature. Tissues were then transferred to a cryoprotective solution for three days at 4°C.

Coronal sections were obtained with a cryostat at 60 μm thickness to improve staining, development, and washing efficacy. Sections were placed in the developing solution for 1 min, washed in double-distilled water for 5 min, dehydrated in alcohol (50, 75, 96, and 100%; 5 min in each dilution), transferred to xylol, and mounted with Entellan (Merck, Darmstadt, Germany).

For morphological analysis, micrographs (20×) at ∼1.0–0.6 mm from Bregma (Khazipov et al., 2015) of the pyramidal neurons from each M1 layer of both hemispheres were obtained from at least three subjects from three different litters per group. To reconstruct the dendritic arbor, only completely impregnated pyramidal neurons, whose dendritic spines and the dendritic arbor could be visualized in different planes, were documented. At least eight neurons per layer (I–II, III–IV, and V–Vl) from three different subjects of different litters per group were analyzed.

For dendritic arbor analysis, a printed image was obtained from the reconstruction of a series of micrographs. Then, dendritic arborization was reconstructed using a magnifying glass (0.25×), a fine-point black marker, and a white sheet placed on each impression by drawing the dendrites from different planes into a single plane. Finally, drawings were scanned to perform the Sholl analysis (Sholl, 1953) and dendritic complexity analysis, using the Fiji software (Schindelin et al., 2012).

For the Sholl analysis, concentric circles were drawn every 10 μM from the soma, and the number of intersections between the increasing circles and the dendritic arbor was quantified. The dendritic complexity index (DCI) was determined by assigning an ordinal value to each of the dendrites, referring to the order in which a branch appears in relation to the first branch (primary dendrite), and applying the following formula (Chameau et al., 2009):


DCI=(ΣOrdinalvalueofdendrite+numberoftotaldendrites)/(numberofprimarydendrites×lengthofdendritearbor)

### Electrophysiology

Two P21 rats from three different litters (*n* = 3) for a total of six animals were used for electrophysiological experiments. Animals were deeply anesthetized with sodium pentobarbital (50 mg/kg, ip) and rapidly decapitated. The brain was quickly removed and placed in an ice-cold sucrose solution saturated with carbogen (95% O_2_/5% CO_2_) containing (in mM): sucrose 210, KCl 2.8, MgSO_4_ 2, Na_2_HPO_4_ 1.25, NaHCO_3_ 26, D-(+)-glucose 10, MgCl_2_ 1, and CaCl_2_ 1, pH 7.2–7.35. After 30–45 s, the hemispheres were separated along the mid-sagittal line, and coronal slices (385 μm thick) containing the M1 were obtained with a vibratome (VT1000S; Leica, Nussloch, Germany). Slices were incubated at 34°C for 30 min and at least for 60 min at room temperature in an incubation solution containing (in mM): NaCl 125, KCl 2.5, Na_2_HPO_4_ 1.25, NaHCO_3_ 26, MgCl_2_ 4, D-(+)-glucose 10, and CaCl_2_ 1, pH 7.4.

Individual slices were transferred to a submersion recording chamber and placed on an upright Nikon FN1 microscope (Nikon Corporation, Minato, Tokyo, Japan). The slices were continuously perfused with artificial cerebrospinal fluid (aCSF) at a constant flow rate (2-3 ml/min) with a peristaltic pump (120S, Watson-Marlow, Wilmington, MA, United States). The aCSF composition was (in mM): NaCl 125, KCl 2.5, Na_2_HPO_4_ 1.25, NaHCO_3_ 26, MgCl_2_ 2, D-(+)-glucose 10, and CaCl_2_ 2; pH 7.4, saturated with carbogen.

Coronal slices containing pyramidal neurons from layers V/VI of M1 were visualized using a Nikon FN-S2N microscope (Nikon, Tokyo, Japan) coupled to an infrared differential interference contrast coupled camera. Borosilicate pipettes were obtained with a horizontal puller (Flaming-Brown P-97, Novato, CA, United States) with a final resistance of 3–6 MΩ when filled with an intracellular solution containing (in mM): potassium gluconate 135, KCl 10, EGTA 1, HEPES 10, Mg-ATP 2, NA_2_-GTP 0.4, and phosphocreatine 10; pH 7.2–7.3 and 290–300 mOsm/L). Whole-cell, patch-clamp recordings were performed with an Axopatch 200B amplifier (Molecular Devices, San José, CA, United States), digitized at a sampling rate of 10 kHz, and filtered at 5 kHz with a Digidata 1322A (Axon Instruments, Palo Alto, CA, United States). Electrophysiological signals were acquired and analyzed offline with pCLAMP10.6 software (Molecular Devices).

To analyze intrinsic membrane properties and neuronal excitability, current-clamp experiments were performed under basal conditions and in the presence of HA (1, 10, and 100 μM). Resting membrane potential (RMP) was determined immediately after the initial break-in from gigaseal to whole-cell configuration. Once RMP was stabilized, a series of negative and positive current steps from −300 pA to rheobase (1 s duration, 30 pA increments) were injected to determine input resistance (Rin), membrane time constant (τ), and the rheobase current. The input resistance (Rin) was determined as the slope of a linear function fitted to the current–voltage (I–V) relationship near the RMP. The membrane time constant (τ) was determined by fitting an exponential function to a voltage response elicited by the injection of a pulse of negative current (−30 pA). The amplitude of the “sag” conductance, defined as the voltage drop in response to a hyperpolarizing current step, was determined by injecting a pulse of −300 pA. Firing frequency was evaluated by applying current pulses from 0 to 500 pA (1 s duration, 50 pA increments) and quantifying the number of evoked action potentials. The rheobase was calculated as the current required to evoke at least one action potential.

Four P21 rats from three different litters (*n* = 3) per group were used to evaluate the effect of the H_1_R antagonist/inverse agonist mepyramine (1 μM) or the H_2_R antagonist/inverse agonist tiotidine (1 μM) on firing frequency in response to the injection of a current step of 300 pA (1 s). Each neuron was registered under the following conditions: (a) no drugs (basal); (b) in the presence of mepyramine or tiotidine; and (c) in the presence of mepyramine or tiotidine and 10 μM HA.

For the electrophysiology experiments, a maximum of two slices per animal and only one neuron per slice were recorded to avoid biased interpretations.

### Depolarization-Evoked [^3^H]-Glutamate Release From Striatal Slices

Glutamate release was performed as previously reported (Giuliani et al., 2011; Casas et al., 2013) in cross-chopped P21 striatal slices (250 × 250 μm) obtained from four pups from the same litter for a total of seven litters per group (*n* = 7). Striatal slices were placed in ice-cold Krebs-Henseleit (KH) solution containing (in mM): NaCI 126, KCI 3, MgSO_4_ 1, KH_2_PO_4_ 1.2, NaHCO_3_ 25, D-glucose 11, pH 7.4 after saturation with O_2_/CO_2_, 95:5% v:v). CaCl_2_ was omitted to reduce excitotoxicity.

Thereafter, slices were equilibrated in KH solution containing 1.8 mM CaCl_2_ for 30 min at 37°C, changing the solution every 10 min. Slices were then transferred to 1 ml KH solution containing 100 nM [^3^H]-glutamate (Perkin Elmer, Boston, MA, United States) in the presence of dihydrokainic acid and aminooxyacetic acid (200 μM each) to prevent [^3^H]-glutamate uptake by glial cells and degradation by transaminases, respectively.

After loading, slices were washed three times with KH solution and randomly distributed (30 μl of sedimented slices) in a perfusion chamber system (Brandel 2500, Gaithersburg, MD, United States) and perfused (1 ml/min) with KH solution for 20 min. Two basal fractions were collected (1 ml each), and [^3^H]-glutamate release was stimulated at the third fraction by switching to a KH solution containing 50 mM KCl (equimolarly substituted for NaCl). The perfusion was returned to standard KH solution and seven fractions were further collected. The Ca^2+^-dependence of [^3^H]-glutamate release was tested in KH solution without CaCI_2_ added.

At the end of the perfusion, the tissue was recovered and treated with 1 ml HCI (1 N) for 1 h, and the tritium content was determined by scintillation counting. Fractional release values were normalized to the fraction collected immediately before fraction 3, and the area under the curve was obtained to determine total [^3^H]-glutamate release.

#### [^3^H]-Mepyramine Binding Assay

The frontal cortex from one P21 rat from 5–6 different litters (*n* = 5–6) was dissected at ∼2.8 to −0.4 mm from Bregma (Khazipov et al., 2015). The tissue was homogenized in a hypotonic solution (10 mM Tris–HCI, 1 mM EGTA, pH 7.4) and centrifuged (20,000 × *g* at 4°C for 20 min). The resulting pellet (membranes) was resuspended in incubation buffer (50 mM Tris–HCI, pH 7.4) and sonicated (3×, 5 s). Binding assays were performed as described in detail elsewhere (Soria-Jasso et al., 1997). Briefly, membrane aliquots were incubated for 60 min at 30°C in 100 μl incubation buffer containing a nearly saturating concentration (10 nM) of the selective H_1_R antagonist [^3^H]-mepyramine (PerkinElmer, Waltham, MA, United States). The incubation was stopped by filtration through Whatman GF/B glass microfiber filters (GE Healthcare Life Science, Marlborough, MA, United States), pre-soaked for 2 h in 0.3% polyethylenimine (PEI), and the radioactivity retained in the filters was determined by scintillation counting.

Non-specific binding was defined in the presence of unlabeled mepyramine (10 μM). Specific binding was calculated by subtracting non-specific binding from total binding and then normalized to the amount of protein per sample determined by the BCA method.

### Enzyme-Linked Immunosorbent Assay (ELISA)

Histamine content was determined by ELISA (cat. 17-HISRT-E01-RES, sensitivity 0.2 ng/ml, 100% HA specificity). The frontal cerebral cortex (∼2.8 to −0.4 mm from Bregma; Khazipov et al., 2015) from one animal of different litters for a total of 5–9 litters per group (*n* = 5-9).

Following the protocol recommended by the supplier (ALPCO R immunoassays, Salem, NH, United States), the tissue was homogenized in 100 μl ice-cold PBS (Polytron PT 2100 Homogenizer, Kinematica, Luzern, Switzerland), centrifuged (10,500 × *g* for 5 min), and the supernatant was recovered to determine per duplicate the HA content using a reference curve constructed with 0, 0.5, 1.5, 5, 15, and 50 ng/ml HA and by determining the absorbance at 450 nm in a multiple detection system (Glomax, Promega, Madison, WI, United States). Values were corrected by protein content determined by the Bradford method (Bradford, 1976).

## Results

### Effect of Chlorpheniramine Treatment on the Expression and Localization of SATB2, FOXP2, and TBR1 in Neonates and 21-Day-Old Pups

H_1_R blockade by the systemic or intrauterine administration of chlorpheniramine at E12 leads to a reduction in FOXP2^+^ deep-layer neurons in the dorsal telencephalon at E14. The cells that preferentially express the H_1_R in the developing cortex are NSCs located in the ventricular zone (Supplementary Figure 1). As a first approach, we determined the expression of *Foxp2* together with two other cortical markers (Satb2 and Tbr1) by qRT-PCR. We found a reduction in the relative expression of Satb2 and Foxp2 to 21 and 56% of control values, respectively, in the frontal cerebral cortex of neonates from chlorpheniramine-treated rats, while Tbr1 relative expression was not affected (Figure 1B).

WB analysis confirmed the lack of effect on TBR1, as the protein level analysis did not show any significant difference with the control group. In contrast to qRT-PCR analysis, this approach showed increased SATB2 and FOXP2 levels in the frontal cortex of P0 neonates of chlorpheniramine-treated rats (240 and 250% of control neonates, respectively; Figure 1C). In accordance with WB, the global immunofluorescence analysis (from the pial to the deepest layer) in M1 showed that there was no effect on TBR1, whereas the fluorescence signals for SATB2 and FOXP2 increased to 180 and 340% of control values, respectively (Figure 1D).

For the layer distribution analysis, the cortex was divided into upper (I and II-IV) and deeper (V/VI) layers, taking into account that layers V/VI represent ∼50% of the total area in the motor cortex of the rat. Our results showed that in neonates of the control group, SATB2 was only expressed in M1 layer I, while FOXP2 and TBR1 were expressed in both upper and deeper layers (Figure 1D), as previously reported for P0 rats (Valle-Bautista et al., 2020). Indeed, the transcription factor immunostaining after birth in the rat is different from the adult cortex and other cortical areas in P0 mice (Huang et al., 2013; Srivatsa et al., 2014). Furthermore, SATB2 did not show the ‘classical’ nuclear immunostaining described for transcription factors (Figure 1D).

The offspring exposed to chlorpheniramine during embryo development showed changes in the distribution of SATB2 and FoxP2, whose location extended ventrally to layer III and throughout all layers, respectively (Figure 1D).

In P21 pups, the layers were identified by their marker distribution in the control animals, as previously reported (Watakabe et al., 2012; Kast et al., 2019). We took as reference the FOXP2 mark to delimit layers V/VI, and SATB2 for upper layers. Although layer IV in the M1 mouse is not visually appreciated, it is present in the rat, but is thinner than in other cortical areas. Hence, we consider this layer a SATB2 expressing area as reported in the literature (Skoglund et al., 1997; Kast et al., 2019; Valle-Bautista et al., 2020). The SATB2, FOXP2, and TBR1 immunostaining were localized as expected in the control group (Figure 2B). In contrast, in pups from chlorpheniramine-treated rats, SATB2 neurons were preferentially observed in layers V and VI. In turn, FOXP2 and TBR1 transcription factors were dorsalized, with FOXP2 located from layers II to VI and TBR1 from layers IV to VI (Figure 2B). Furthermore, in the offspring of chlorpheniramine-treated rats, the total fluorescence increased significantly to 177, 178, and 141% of the control group for SATB2, FOXP2, and TBR1, respectively (Figure 2C).

### Effect of Chlorpheniramine on the Dendritic Arbor of Pyramidal Neurons of 21-Day-Old Offspring

**FIGURE 3:**
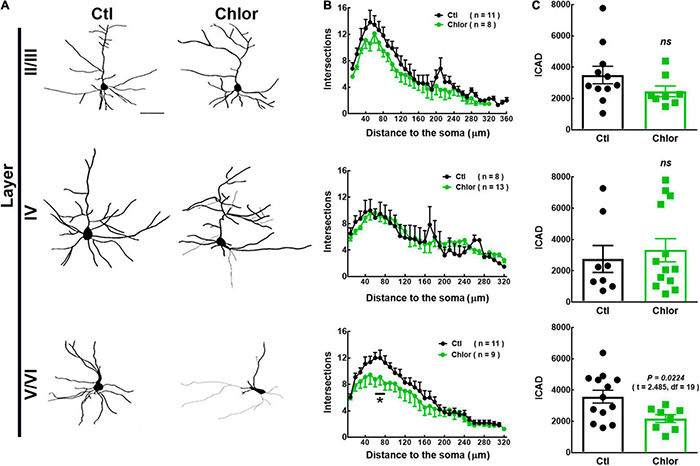
Analysis of the dendritic arbor in 21-day-old offspring from control and chlorpheniramine-treated rats. **(A)** Representative reconstruction of Golgi-Cox-stained projection neurons in cortical layers I–VI. **(B)** Sholl analysis. Values are means ± SEM from the number of dendrite intersections with respect to the distance from the soma in Control (Ctl, black dots) and Chlorpheniramine (Chlor, green dots) groups. *P < 0.5 versus control values at ∼60 and ∼80 μM; multiple *t*-test. **(C)** Analysis of the dendritic complexity index (DIC) of neurons obtained from the offspring of control (black bars) and chlorpheniramine (green bars) groups (*n* = 3–4; P21). The statistical analysis was performed with unpaired Student’s *t*-test. *ns, non-significant*.

To analyze the morphology of M1 pyramidal neurons in P21 pups, reconstructions of the neurons stained with the Golgi-Cox were performed (Figure 3A). Compared to the control group, the systemic administration of chlorpheniramine from E12 to E14 promoted significant changes exclusively in deep-layer neurons of P21 pups, with a significant reduction in the number of proximal processes at ∼60 and ∼80 μM from the soma, as well as in the DCI (Figures 3B,C).

### Effect of Chlorpheniramine on M1 Deep-Cortical Layer Neuron Function in 21-Day-Old Offspring

**FIGURE 4:**
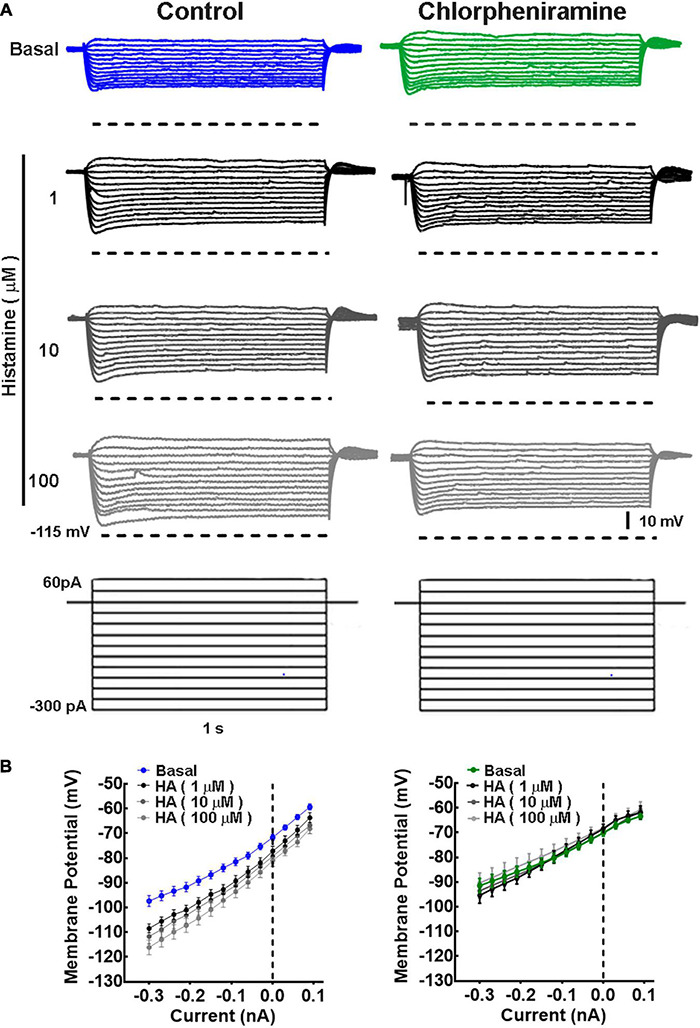
Current/voltage relationship in deep cortical primary motor cortex neurons of 21-day-old offspring from control and chlorpheniramine-treated dams. **(A)** Representative traces of the changes in the membrane potential induced by a series of positive and negative current injections (1 s, 30 pA increments from −300 to 60 pA) from deep cortical layer neurons of Control (blue) and chlorpheniramine (green) groups, in the absence (basal, upper traces) and presence of the indicated concentrations of histamine (lower traces in grayscales). **(B)** Quantitative analysis. Values are means ± SEM from nine neurons for each condition from 6 P21 animals from three different litters per group. The line is the best-fit linear regression for the control (blue) chlorpheniramine (green) groups under basal conditions and in the presence of histamine (grayscales). Values for the passive electrophysiological properties are given in Table 2.

**FIGURE 5:**
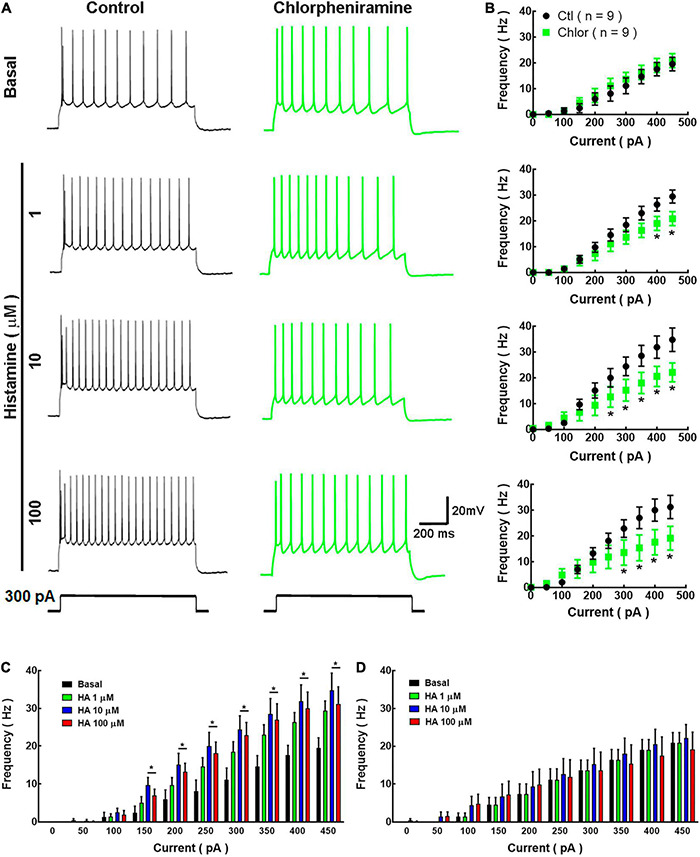
Analysis of action potential frequency in deep cortical neurons. **(A)** Representative traces of the action potentials generated by a depolarizing pulse (300 pA, 1 s) in the absence and presence of histamine (1, 10, and 100 μM) in deep cortical neurons of the offspring of Control (Ctl; black) and chlorpheniramine (Chlor; green) groups. **(B)** Current-frequency relationship. Values indicate the action potential frequency (Hz) induced by depolarizing currents (0–450 pA) and are means ± SEM from three independent experiments. *P < 0.05 when compared with the control group, Repeated Measures ANOVA and Sidak’s test. **(C,D)** Histamine effect on the current-frequency relationship in deep cortical neurons from the control and chlorpheniramine groups, respectively. Values are means ± SEM from nine neurons for each condition from 6 P21 animals from three different litters per group. *P < 0.05 compared with the corresponding basal value; one-way ANOVA and Dunnett’s multiple comparisons test.

**FIGURE 6:**
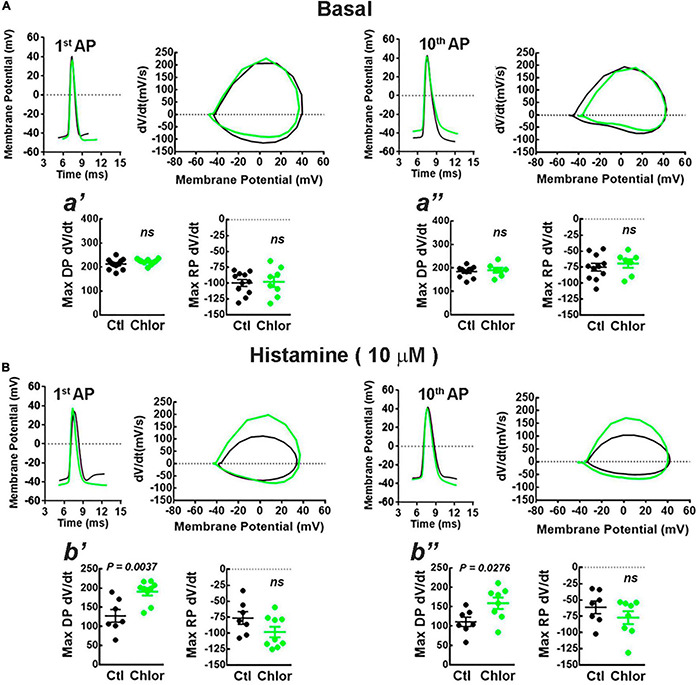
Phase plot analysis of the action potentials. **(A,B)** Representative action potentials induced by the injection of depolarizing current (300 pA for 1 s) (left) and the corresponding phase plots (right) for the first (1st AP) and 10th (10th AP) action potentials from deep layers cortical neurons of the offspring of control (Ctl; black) and chlorpheniramine-treated (Chlor; green) dams in the absence **(A)**, (basal) or presence of 10 μM histamine **(B)**. The graphs below (a′, a′′, b′, and b′′) show the analyses of the maximum rate of the depolarization (Max DP dV/dt) and repolarization (Max RP dV/dt) phases of the 1st AP (a′ and b′) and 10*^th^* AP (a′′ and b′′) under basal condition (a′ and a′′) or histamine stimulation (b′: Max DP dV/dt: *t* = 3.48, df = 11; b′′: Max DP dV/dt: *t* = 2.48, df = 13) for the Control (Ctl; black) and chlorpheniramine (Chlor; green) groups. Values are means ± SEM from 7 to 11 neurons for each condition of 6 P21 animals from three different litters per group. The statistical analysis was performed with unpaired Student’s *t*-test. *ns, non-significant*.

Since morphological changes were observed in M1 deeper layers, we determined the electrophysiological properties in pyramidal neurons from these layers. Under basal conditions, no differences between groups were observed in the neuronal passive and active electrophysiological properties (Figures 4–6). However, while HA increased the membrane resistance (Rin) in the control group, in the chlorpheniramine group this parameter was significantly lower compared to the control group and was not affected by HA (Table 2).

**TABLE 2:** Electrophysiological properties of pyramidal neurons in layers V/VI of the primary motor cortex of 21-day-old pups of control and chlorpheniramine-treated rats.

		Control (*n* = 9)				Chlorpheniramine (*n* = 9)		
	Basal	Histamine (μM)			Basal	Histamine (μM)		
		1	10	100		1	10	100
RMP (mV)	−69.1 ± 1.1	−68.5 ± 0.9	−68.5 ± 0.5	−66.1 ± 1.6	−71.2 ± 0.8	−70.6 ± 0.7	−69.7 ± 0.7	−68.7 ± 2.4
Rin (MΩ)	100.2 ± 5.3	114.2 ± 3.7	118.5 ± 4.3^*^	126.8 ± 6.7^***^	92.4 ± 9.0	95.4 ± 12.7*^a^*	97.0 ± 15.6*^b^*	94.8 ± 17.2*^c^*
τ (ms)	14.8 ± 1.2	18.2 ± 0.8	16.8 ± 1.4	16.4 ± 2.0	11.8 ± 1.1	12.9 ± 2.8	12.0 ± 1.9	10.4 ± 1.8*^a^*
Rheobase (pA)	172.2 ± 20.6	133.3 ± 11.8	155.5 ± 13.0	133.3 ± 11.7	188.8 ± 37.0	188.8 ± 27.3*^a^*	211.1 ± 37.9*^a^*	227.7 ± 50.7*^c^*

Passive membrane properties were obtained from experiments illustrated in Figure 4. The membrane time constant (τ) was obtained at −30 pA. Differences within the same group were evaluated with repeated-measures two-way ANOVA and post hoc Dunnett’s multiple comparison test. *P < 0.05, ***P < 0.001 versus basal. Differences between groups were analyzed with Mixed ANOVA of Repeated Measures followed by Sidak’s test. ^a^P < 0.05, ^b^P < 0.01, ^c^P < 0.001 versus control.

In basal conditions, the firing frequency did not differ between groups (Figures 5A,B). However, and as expected, HA (1, 10, and 100 μM) increased the firing frequency in neurons from the control group, with significant effect from 150 pA and at 10 and 100 μM (Figure 5C). In contrast, HA did not alter the firing frequency of neurons from the chlorpheniramine group (Figure 5D).

Phase plots from the first and tenth action potentials elicited by a current injection of 300 pA revealed that under basal conditions the conductances underlying the depolarization (Na^+^) and repolarization (K^+^) phases of the action potential were not different in neurons from the offspring of chlorpheniramine-treated rats. However, in the presence of 10 μM HA, the maximum rate of the depolarizing phase increased in these neurons (Figure 6), suggesting changes in either the expression or the biophysical properties of fast-inactivating Na^+^ currents.

HA increases neuronal excitability through H_1_R activation (McCormick and Williamson, 1991; Ji et al., 2018; Zhuang et al., 2018). Therefore, H_1_R density was determined in P21 frontal cortex membranes from both groups. Unexpectedly, the products of chlorpheniramine-treated dams showed increased H_1_R density compared to the control group (69 ± 7 and 42 ± 7 fmol/mg protein, for the control and chlorpheniramine groups, respectively; *P* = 0.0046; *t* = 3.74, df = 9, unpaired *t*-test; control *n* = 6 and chlorpheniramine *n* = 5). Likewise, an increase in cortical HA content was observed (826 ± 278 and 260 ± 30 ng/mg protein for the control and chlorpheniramine groups, respectively; *P* = 0.0170; *t* = 2.76, df = 12, unpaired *t*-test; control *n* = 9 and chlorpheniramine *n* = 5).

The increase in H_1_R density and HA content in pups from chlorpheniramine-treated rats is counterintuitive with the decrease in HA-induced excitability. Moreover, the increase in neuronal firing observed in neurons from animals of the control group could also involve H_2_R activation (Panula et al., 2015). Therefore, the participation of each receptor was evaluated by determining the firing frequency at a depolarizing pulse of 300 pA in the presence of the H_1_R antagonist/inverse agonist mepyramine (1 μM) or the H_2_R antagonist/inverse agonist tiotidine (1 μM) and HA (10 μM).

**FIGURE 7:**
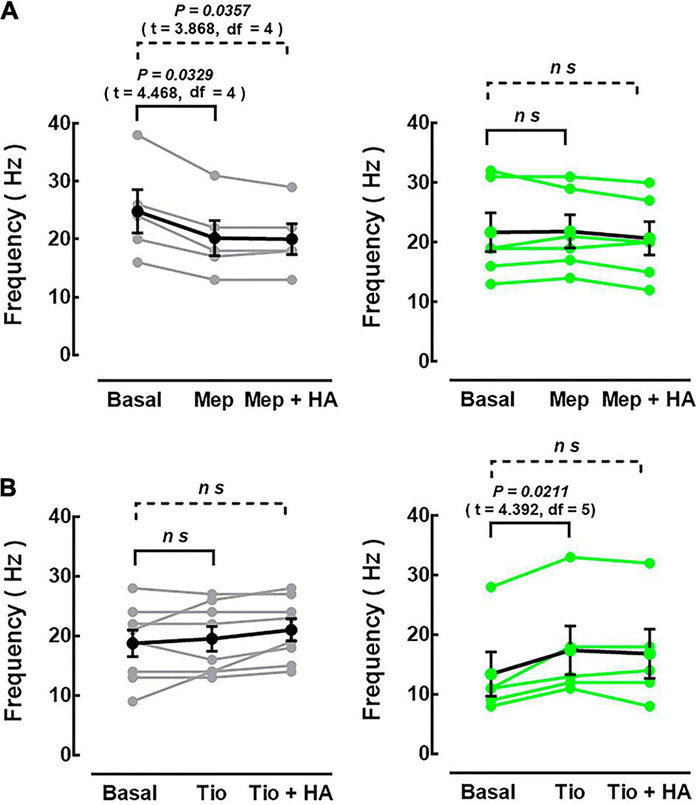
Effect of antagonists/inverse agonists at histamine H_1_ or H_2_ receptors on action potential frequency. The firing frequency induced by the injection of depolarizing current (300 pA, 1 s) in neurons from the control [**(A,B)**, left black and gray circles] and chlorpheniramine [**(A,B)**, right green circles] groups was evaluated in basal conditions (no drugs), after the perfusion of the H_1_R antagonist/inverse agonist mepyramine (Mep, 1 μM) or the H_2_R antagonist/inverse agonist tiotidine (Tio, 1 μM), and in the presence of histamine (10 μM) and mepyramine (Mep + HA) or tiotidine (Tio + HA). Values (control: black dots; chlorpheniramine: green dots) are means ± SEM from 5 to 8 neurons for each condition of 4 P21 animals from three different litters per group. Black or green lines connect values from the same neuron. The statistical analysis was performed with one-way ANOVA and Holm-Sidak’s multiple comparisons test. *ns, non-significant*.

In neurons from animals of the control group, mepyramine decreased basal firing frequency from 24.80 ± 3.72 to 20.20 ± 3.05 Hz and prevented the increase in firing induced by HA (20.00 ± 2.66 Hz). In contrast, tiotidine had no effect on its own but prevented the HA-induced increase in firing frequency (18.75 ± 2.23, 19.5 ± 2.07, and 21 ± 1.87 Hz for basal, tiotidine, and tiotidine + HA, respectively; Figures 7A,B). In neurons from animals of the chlorpheniramine group, mepyramine did not modify the basal firing frequency (21.67 ± 3.24, 21.83 ± 2.76, and 20.67 ± 2.81 Hz in basal, mepyramine, and mepyramine + HA conditions, respectively). However, it prevented the HA effect for basal, tiotidine, and tiotidine + HA conditions (12.17 ± 3.26, 15.17 ± 3.42, and 14.67 ± 3.98, respectively; Figures 7A,B). Tiotidine alone promoted a significant increase in the firing frequency in the basal condition and prevented the HA-induced increase (Figures 7A,B).

**FIGURE 8:**
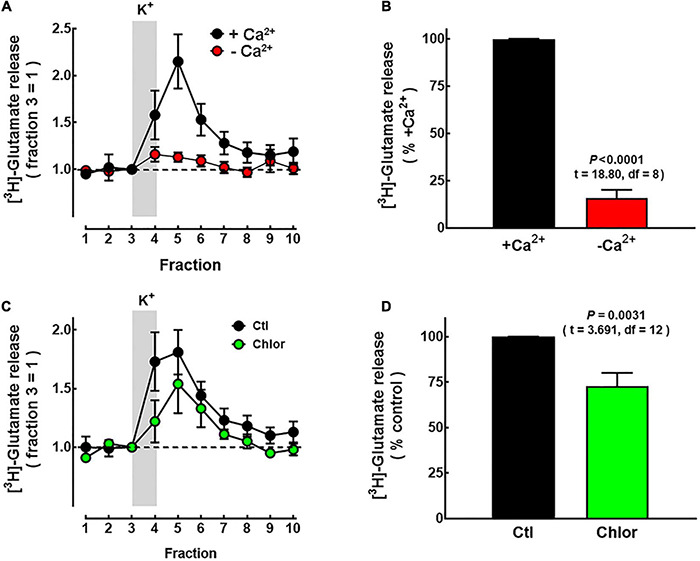
Effect of chlorpheniramine administration to pregnant rats on [^3^H]-glutamate release in striatal slices from 21-day-old offspring. Slices of P21 animals were obtained as described in Methods and perfused with Krebs–Henseleit (KH) solution. The release of [^3^H]-glutamate was evoked by raising the concentration of K^+^ in the perfusion solution from 4 to 50 mM for the period indicated by the vertical gray bar. **(A)** Calcium-dependence of [^3^H]-glutamate release. Representative experiment of slices perfused with KH solution with no CaCl_2_ added (red circles) or containing 1.8 mM CaCl_2_ (black circles). Values were normalized to the release in fraction 3 (equals 1) and represent means ± SEM from striatal pools of four P21 animals. **(B)** Analysis of the area under the curve after subtraction of the basal release. Values are expressed as percentage of the release from slices exposed to Ca^2+^ and are means ± SEM from striatal pools of four P21 animals per litter from five different dams per condition. **(C)** Representative experiment for depolarization-evoked [^3^H]-glutamate release from striatal slices of P21 offspring from Control (Ctl; black circles) and chlorpheniramine-treated rats (Chlor; green circles). Values are means ± SEM from striatal pools of four P21 animals per group. **(D)** Analysis of the area under the curve after subtraction of basal release. Values are expressed as percentage of the release from slices of control animals in the same experiment and are means ± SEM from striatal pools of four P21 animals per litter from seven different dams per condition. The statistical analysis was performed with unpaired Student’s *t*-test.

To further evaluate the functionality of M1 deep cortical neurons, which project to the striatum, K^+^-evoked [^3^H]-glutamate release from striatal slices was evaluated. The release of the labeled neurotransmitter evoked by 50 mM K^+^ was markedly dependent on the presence of Ca^2+^ ions in the perfusion medium (84.3 ± 5.6% of total release, *P* < 0.0001; *t* = 18.80, df = 8, unpaired Student’s *t*-test; five experiments; Figures 8A,B). K^+^-evoked [^3^H]-glutamate release was significantly lower in slices from the offspring of chlorpheniramine-treated rats (72.7 ± 7.3% of the release in slices from animals of the control group; Figures 8C,D).

## Discussion

The main findings of this work are that the systemic administration of the H_1_R antagonist/inverse agonist chlorpheniramine to pregnant rats (from E14 to E16) resulted in postnatal effects on transcription factor expression and distribution, decreased deep-layer dendritic arborization and excitability, and diminished depolarization-evoked [^3^H]-glutamate release from striatal slices.

To our knowledge, there is no direct evidence that chlorpheniramine permeates the feto-placental barrier. However, its high membrane permeability, moderate protein binding, rapid distribution in various tissues (including the brain), and high tissue/plasma concentration ratios (Huang and Chiou, 1981; Sakurai et al., 1992; Koch et al., 1998; Mahar Doan et al., 2004; Xu et al., 2018) support that chlorpheniramine reaches the fetal circulation. This notion is further supported by previous studies showing chlorpheniramine effects similar to those reported here on neuronal markers during brain development by the *in utero* or systemic administration of the drug (Molina-Hernández et al., 2013; Solís et al., 2017).

The changes reported here could be attributed to the indirect effect of chlorpheniramine on H_1_Rs in the placenta, where it has an angiogenic function (Moya-García et al., 2021), and H_1_R blockade may thus lead to negative consequences on fetoplacental circulation. However, the results reported here, together with the effect of HA and chlorpheniramine on neurogenesis *in vivo* and *in vitro* reported previously (Molina-Hernández and Velasco, 2008; Molina-Hernández et al., 2013; Solís et al., 2017), support a direct effect on brain development.

Chlorpheniramine also acts as an antagonist at muscarinic receptors (M1–M5), and the effects reported here could also involve the blockade of such receptors. However, chlorpheniramine binds to muscarinic receptors with very low affinity (IC_50_ 17–78 μM; Yasuda and Yasuda, 1999), contrasting with its high affinity for H_1_Rs (K_*i*_ 1–3 nM; Arias-Montaño and Young, 1993; Hishinuma et al., 2017). Furthermore, the expression of muscarinic receptors in rat cerebral cortex begins at E16 (Ma et al., 2000; Li et al., 2001), making therefore unlikely a chlorpheniramine effect at muscarinic receptors during the period of administration explored here (E12–E14). Hence, we can assume that the chlorpheniramine effects reported here are due to the antagonism of H_1_Rs expressed in the cortical neuroepithelium NSCs (Supplementary Figure 1).

As previously mentioned, HA *via* H_1_R activation affects FOXP2 phenotype *in vivo* and *in vitro* during embryo differentiation (Molina-Hernández et al., 2013), whereas the long-term effects of blocking H_1_Rs during early cortical neurogenesis resulted not only in changes in FOXP2 expression and distribution but also in SATB2 and TBR1 expression and localization.

The higher protein levels of FOXP2 and SATB2 in neonates and of FOXP2, SATB2, and TBR1 in P21 pups from chlorpheniramine-treated rats, suggest compensatory mechanisms for the reduction in neurogenesis promoted by H_1_R blockade, which may lead to an increase in neuron generation after the chlorpheniramine treatment. Therefore, it would be important to evaluate other neuronal and cell proliferation markers during and after chlorpheniramine administration.

The increased FOXP2 and SATB2 protein levels showed discrepancies with the corresponding mRNA relative expression at P0. These differences could be related to enhanced degradation or lower transcription of their mRNAs induced by the high levels of the protein, that might involve microRNAs, changes in RNA processing, protein modifications that increase the half-life, and negative feedback loops induced by high protein levels that repress transcription (Pan et al., 2006; Sheehan-Rooney et al., 2010; Singh, 2011; Clovis et al., 2012; Bornstein et al., 2014; Fu et al., 2014). Of note, in the Western blot analysis of SATB2, bands with lower molecular weight were detected in samples from neonates, which in conjunction with the absence of nuclear mark for the transcription factor suggest a high rate of SATB2 degradation.

The increase in SATB2 and FOXP2 in P0 and later in TBR1 in P21 pups of chlorpheniramine-treated rats reported here suggest changes in neuron pattern lamination and the establishment of neuronal networks, probably due to a dysregulation of deep-layer cortical neurogenesis during embryo development. The above is supported by studies indicating the importance of these transcription factors in cell migration, differentiation, neuron layer specification, and/or cortical axonal pathways (Bedogni et al., 2010; Tsui et al., 2013; Devanna et al., 2014; Leone et al., 2015).

FOXP2 regulates NSCs transition to intermediate progenitor cells (IPCs) in the ventricular zone. The reduction of FOXP2 translation through short hairpin RNA increases NSCs at the expense of IPCs, reducing neuron differentiation (Tsui et al., 2013). Thus, in chlorpheniramine-exposed embryos, the fraction of self-renewed NSCs increases due to H_1_R blockade, reducing neuron differentiation temporarily. Once the drug is cleared, IPCs-mediated increase in neurogenesis results in higher postnatal FOXP2, SATB2, and TBR1 levels. Interestingly, increased postnatal FOXP2 may affect neurite outgrowth, synaptic plasticity, and the development of cortico-striatal, cortico-thalamic, and cortico-cerebellar circuits, and FOXP2 mutations impair cognition, language, and the ability to learn the movements required for speech. Individuals with one functional copy of the gene show a deterioration of expressive and receptive language related to cortical, striatal, and cerebellar impairments (Vargha-Khadem et al., 2005; Vernes et al., 2011; den Hoed et al., 2021). In mice, FOXP2 is involved in intra-species communication, and heterozygous animals show reduced ultrasonic vocalization and impaired movement (Shu et al., 2005).

Although the effect of FOXP2 over-expression has not been assessed, our results suggest that an increase in FOXP2 promotes changes in the dendritic arborization of deep-layer neurons and ultrasonic vocalization performance and movement. Reduced methamphetamine-stimulated motor activity in male and female offspring from pregnant rats treated with chlorpheniramine has been reported recently by our group. Interestingly, only males showed reduced speed movement under basal conditions, suggesting sex-specific alterations (Márquez-Valadez et al., 2019), this being the reason why only male animals were included in the present study. However, it will be important to assess females in future studies.

Leone et al. (2015) showed that SATB2 is expressed by cortico-cortical and cortico-striatal layer V neurons in M1, a pattern also observed here. SATB2 is widely expressed in the adult mouse CNS, including the cerebral cortex (Huang et al., 2013). We previously reported that the layer distribution of this transcription factor in M1 at P0 rats differs from the adult cortex, with very few cells presenting a clear nuclear mark, suggesting a distinct or no function for SATB2 in early postnatal life (Valle-Bautista et al., 2020). This transcription factor plays an essential role in the neural development and function of the CNS; however, the mechanisms involved remain to be fully understood. SATB2 appears to participate as a factor that defines the upper and deeper layers by regulating the expression of other transcription factors essential for layer specification such as Fezf2, Ctip2, Sox5, and Tbr1 (Greig et al., 2013; McKenna et al., 2015).

Furthermore, SATB2 regulates the development of callosal projections, as well as the cortico-thalamic and cortico-striatal pathways (Srinivasan et al., 2012; Sohur et al., 2014; Leone et al., 2015). Interestingly, SATB2-deficient neurons fail to form the corpus callosum and project instead to subcortical areas (Alcamo et al., 2008). Although neuronal pathways were not determined here, the alterations in transcription factor distribution and protein levels observed in the M1 of P21 offspring of the chlorpheniramine-treated dams suggest that the products will present vast affection in the axonal pathways linking both hemispheres and projecting to subcortical areas, in addition to the impaired neuron distribution reported in this work. Furthermore, in Satb2 knockout mice, FOXP2 is absent from layers V and VI (Leone et al., 2015), suggesting that increased SATB2 mark may contribute to a higher protein level of FOXP2 and, probably, of TBR1 as well. Experiments in which SATB2 expression is abolished or overexpressed postnatally will shed light on this matter. Furthermore, the impaired distribution and increased fluorescence levels of TBR1 at P21 could be a consequence of SATB2 changes in the chlorpheniramine-treated group, since these alterations appeared earlier than those observed for TBR1. This hypothesis is supported by Tbr1 knockout mice, which display corpus callosum agenesis (Hevner et al., 2001), likewise SATB2-deficient animals (Alcamo et al., 2008). Moreover, TBR1 participates in frontal identity and is essential for the specification and projection patterns of layer VI cortico-thalamic neurons (Hevner et al., 2002; Bedogni et al., 2010), in which SATB2 is also involved (Srinivasan et al., 2012; Sohur et al., 2014; Leone et al., 2015).

Moreover, the changes in the distribution of the transcription factors in the M1 of offspring of chlorpheniramine-treated dams suggest an altered migration, an aspect that need further studies.

Mainen and Sejnowski (1996) reported that the dendritic structure influences the firing pattern of neocortical neurons. As we observed changes in the DCI of deep-layer cortical neurons, the passive and active properties of these neurons were studied. Our electrophysiological experiments showed that HA reduced membrane resistance in cortical deep-layer neurons from the chlorpheniramine group (Table 2). These results suggested modulation of K^+^ conductances active near the resting membrane potential. However, phase-plot analysis showed that HA increases Max DP dV/dt in the 1*^st^* and 10*^th^* action potentials, which can be attributable to enhanced Na^+^ conductances.

Na^+^ currents shape the rising phase of the action potential (de Haas and Vogel, 1989), and it would thus be expected that the increased Na^+^ influx through voltage-gated Na^+^ channels (Na_*v*_) enhances neuronal excitability. However, it has been reported that through a negative feedback loop an increase in Na^+^ conductance reduces the firing rate when a large current is injected, promoting the neurons to become less sensitive to changes in excitatory inputs and maintaining the firing rate, most likely by inducing the Na_*v*_ inactive state (Jung et al., 1997; Mickus et al., 1999; Kispersky et al., 2012; Navarro et al., 2020). Therefore, changes in Na_*v*_ kinetics could explain the lower frequency and increased Na^+^ conductance (higher Max DP dV/dt in the 1*^st^* and 10*^th^* action potentials) in neurons from the chlorpheniramine group.

Decreased H_1_R expression or activation could have contributed to the reduced response to HA in the chlorpheniramine group. However, H_1_R density and HA content were higher in the frontal cerebral cortex of the products of chlorpheniramine-treated dams (164 and 317% of values for the control group, respectively). In heterologous expression systems, H_1_R stimulation up-regulates receptor expression through the activation of H_1_R gene transcription (Das et al., 2007), and this mechanism could be triggered by increased HA levels. Yet, M1 neurons from pups of chlorpheniramine-treated dams were unresponsive to HA. This unexpected result could be due to most H_1_Rs lacking coupling to their cognate G proteins leading to reduced receptor functionality. This possibility is supported by the experiments with the H_1_R antagonist/inverse agonist mepyramine, which did not affect the firing frequency in the chlorpheniramine group suggesting lack of H_1_R constitutive activity. In this scenario, the increase in HA content could derive from enhanced synthesis and release to compensate the reduced receptor function. In contrast, the increased frequency induced by the H_2_R antagonist/inverse agonist tiotidine in the chlorpheniramine group is challenging to explain.

Furthermore, neurons can express both H_1_Rs and H_2_Rs; hence, it is likely that the increased response to HA of deep-layer M1 cortical neurons observed in the control group is promoted by the activation of both receptors and that cross-regulation may be induced by high levels of HA or receptor antagonists. In line with this, in native and recombinant systems, both receptors desensitize when cells are exposed to a sustained stimulus with H_1_R or H_2_R agonists. This cross-desensitization depends on G protein-coupled receptor kinase 2 (GRK2), and upon activation H_1_Rs and H_2_Rs can form heteromers and be internalized in endosomes (Alonso et al., 2013).

The dorsal striatum receives glutamate innervation from the M1 deep-layer neurons and thalamus (Parent and Parent, 2006). However, it is likely that the reduced K^+^-evoked [^3^H]-glutamate release observed in striatal slices from the chlorpheniramine group (Figure 8) is mainly due to altered functionality of M1 neurons or a reduction in the density of glutamatergic axons originated in the cerebral cortex. The above is suggested since the cortical glutamatergic input is more abundant than the thalamic input (Lacey et al., 2005; Raju et al., 2006); however, we cannot exclude a reduction in the thalamic glutamatergic innervation.

Dysfunction of the CNS histaminergic system has been related to several basal ganglia disorders, including Parkinson’s disease and Tourette’s syndrome (Bolam and Ellender, 2016; Rapanelli and Pittenger, 2016), as well as other neurological diseases such as autism and Alzheimer’s disease. Transgenic animal models and results previously reported by our group (Mobarakeh et al., 2005; Chepkova et al., 2012; Molina-Hernández et al., 2013; Sundvik et al., 2013; Ambree et al., 2014; Parmentier et al., 2016; Baronio et al., 2018; Márquez-Valadez et al., 2019) provide evidence for a role of H_1_Rs in CNS development.

Antihistamines are commonly used during pregnancy for rhinitis, asthma, allergic conjunctivitis, acute urticaria, anaphylaxis, and food- and drug-allergy treatment. Most antihistamines are considered in FDA category B (no evidence of human risk after *in utero* exposure; Kalpaklioglu and Baccioglu, 2012; Thorpe et al., 2013; Gilboa et al., 2014). However, given their wide use and availability without prescription, a small increase in the risk of specific birth defects may have significant clinical and public health implications. A systemic review and meta-analysis study including 37 studies (33 cohort and 4 case-control studies) on the risk of adverse pregnancy outcomes (spontaneous abortions, prematurity, stillbirth, and low birth weight) after the first trimester of the exposure to H_1_R-antihistamines concludes that there is no association of the use of these drugs with an increased risk of major malformation or other adverse fetal outcomes (Etwel et al., 2016).

In contrast, a positive association between chlorpheniramine during pregnancy with eye and ear defects has been reported (Gilboa et al., 2009). Furthermore, Li et al. (2013) reported no epidemiological link between the most common antihistamines (diphenhydramine, loratadine, doxylamine, and chlorpheniramine) and major congenital malformations. Nonetheless, they also reported a 3-fold risk of chlorpheniramine for ear defects, and odd ratios >1 in the association of this drug to spina bifida and encephalocele.

The chlorpheniramine dose used here was previously reported as non-teratogenic in the rat (Naranjo and de Naranjo, 1968). Moreover, we did not observe any congenital malformation in the offspring of chlorpheniramine-treated rats (data not shown). In contrast, we detected an impaired cortical cytoarchitecture and altered dendritic arborization of deep-layer neurons. Finally, under basal conditions, no physiological changes were observed between groups. All these data suggest that chlorpheniramine (or other H_1_R antagonists) during pregnancy may promote subtle developmental changes, with a clinical relevance on fetal programming of neurological diseases.

## Conclusion

Our data indicate that through H_1_R activation, HA regulates the development of the M1 and that receptor blockade during corticogenesis will alter cortical deep-layer neuron distribution and function. Together with the report by Márquez-Valadez et al. (2019), this study suggests that the use during pregnancy of first-generation antihistamine drugs bears clinical relevance. However, further research is required to establish if the described effects are compensated or intensified in adult and elderly male and female animals. It would also be important to evaluate if the effects observed here are exclusive of the M1 area.

## Data Availability Statement

The original contributions presented in the study are included in the article/Supplementary Material, further inquiries can be directed to the corresponding author.

## Ethics Statement

The animal study was reviewed and approved by Comité Institucional para el Cuidado y uso de Animales de Laboratorio del Instituto Nacional de Perinatología.

## Author Contributions

RV-B performed and analyzed most of the experiments. BM-V conducted and analyzed the release experiments. GH-L, EG, and EJG performed and analyzed the electrophysiology experiments. N-FD, J-AA-M, and AM-H participated in all steps of the study, including conception, critical revision of the manuscript, experimental analysis, and supervision. AM-H approved the final manuscript. All authors participated in data analysis and discussion and drafting of the manuscript.

## Conflict of Interest

The authors declare that the research was conducted in the absence of any commercial or financial relationships that could be construed as a potential conflict of interest.

## Publisher’s Note

All claims expressed in this article are solely those of the authors and do not necessarily represent those of their affiliated organizations, or those of the publisher, the editors and the reviewers. Any product that may be evaluated in this article, or claim that may be made by its manufacturer, is not guaranteed or endorsed by the publisher.
