# Supplementary material for: Long-Term Functional and Cytoarchitectonic Effects of the Systemic Administration of the Histamine H1 Receptor Antagonist/Inverse Agonist Chlorpheniramine During Gestation in the Rat Offspring Primary Motor Cortex
Source: Front Neurosci. 2022 Jan 24;15:740282. doi: 10.3389/fnins.2021.740282 (PMC8820484; doi:10.3389/fnins.2021.740282)
Supplement: Supplementary file 1 [file Data_Sheet_1.docx]

***Supplementary Material***

**Supplementary Figure 1. H_1_ receptor expression in the rat neuroepithelium.** H_1_R immunodetection. Left, coronal view of the telencephalon (E14). The area outlined in the red dotted square corresponds to the area shown in the representative epifluorescence micrograph on the right (20×), which shows H_1_Rs (red), Nestin (green), and DAPI (blue). MZ, marginal zone; SVZ, subventricular zone; VZ, ventricular zone. The white arrows indicate H_1_R positive cells in the VZ.
