# Supplementary figures and images for: Long-Term Functional and Cytoarchitectonic Effects of the Systemic Administration of the Histamine H1 Receptor Antagonist/Inverse Agonist Chlorpheniramine During Gestation in the Rat Offspring Primary Motor Cortex
Source: Front Neurosci. 2022 Jan 24;15:740282. doi: 10.3389/fnins.2021.740282 (PMC8820484; doi:10.3389/fnins.2021.740282)

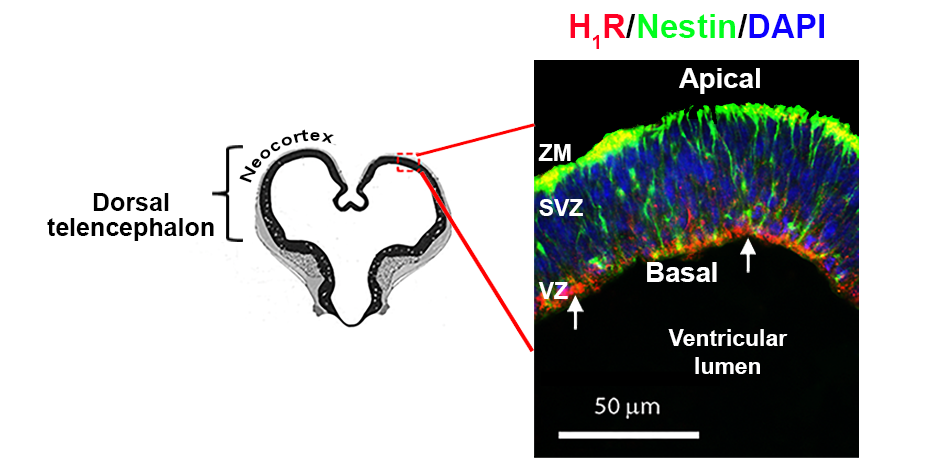

Supplement: Supplementary file 2 [file Image_1.TIF]
